# Recent advances in immunotherapy for cervical cancer

**DOI:** 10.1007/s10147-025-02699-0

**Published:** 2025-01-31

**Authors:** Aiko Ogasawara, Kosei Hasegawa

**Affiliations:** Department of Gynecologic Oncology, Saitama Medical University International Medical Center, 1397-1 Yamane, Hidaka-shi, Saitama 350-1298 Japan

**Keywords:** Cervical cancer, Immune checkpoint inhibitor, Immunotherapy, Advanced cervical cancer, Recurrent cervical cancer, Metastatic cervical cancer

## Abstract

Cervical cancer is the third most common malignant tumor in women worldwide in terms of both incidence and mortality. The field of cervical cancer treatment is rapidly evolving, and various combination therapies are being explored to enhance the efficacy of immune checkpoint inhibitors (ICI) and provide new treatment options for patients at different disease stages. Clinical trials involving immune checkpoint inhibitors are now being conducted following a phase 3 trial with cemiplimab, an ICI, which demonstrated a significant improvement in prognosis in advanced or metastatic cervical cancer patients. These trials include monotherapy and combination therapy with other immune therapies, chemotherapy, or radiation therapy. Furthermore, other approaches for controlling tumors via the immune system, such as therapeutic vaccination for specific tumor antigens or immune cell therapy including chimeric antigen receptor (CAR)-T cell therapy and tumor-infiltrating lymphocytes are being investigated. Ongoing trials will continue to illuminate the optimal strategies for combining these therapies and addressing challenges associated with immune checkpoint failure in cervical cancer. Herein, we conducted a review of articles related to immunotherapy for cervical cancer and describe current treatment strategies for cervical cancer via immunotherapy.

## Introduction

Cervical cancer is the third most prevalent malignant tumor among women globally both in terms of incidence and mortality [1]. Particularly challenging are recurrent or advanced cases that are unresponsive to primary treatments and thus have a bleak prognosis. The introduction of the human papilloma virus (HPV) vaccine on a global scale has demonstrated effectiveness in preventing HPV-related neoplasms [2]. However, regional disparities in vaccine adoption persist, and cervical cancer remains a substantial concern not only in countries where the HPV vaccine is not widely available but also in countries where the vaccine is commonly administered. For example, in the United States, survival rates have shown minimal improvement since the 1970s, and instances of stage IV advanced cervical cancer are increasing [3].

Consequently, novel treatment approaches are urgently required. Although conventional cytotoxic chemotherapy has yielded limited improvements in prognosis, reports on the efficacy of immunotherapy for cervical cancer are increasing. Cervical cancer is often associated with persistent HPV infection, and the HPV oncoproteins E6 and E7 expressed in tumor cells, play a pivotal role in tumorigenesis and the maintenance of malignant characteristics; therefore, these oncoproteins are considered ideal targets for the immune system [4]. This underscores the potential of targeting HPV via immunotherapies, such as antibodies and therapeutical vaccines, and HPV-related cancers via T-cell therapies. Numerous encouraging prospects exist for harnessing the immune system in treating cervical cancer [5]. This review aims to explore recent advances and evidence in immunotherapy, including the use of immune checkpoint inhibitors (ICIs), various immune-related therapies, and combination therapies for cervical cancer.

## Tumor microenvironment in cervical cancer

**Fig. 1:**
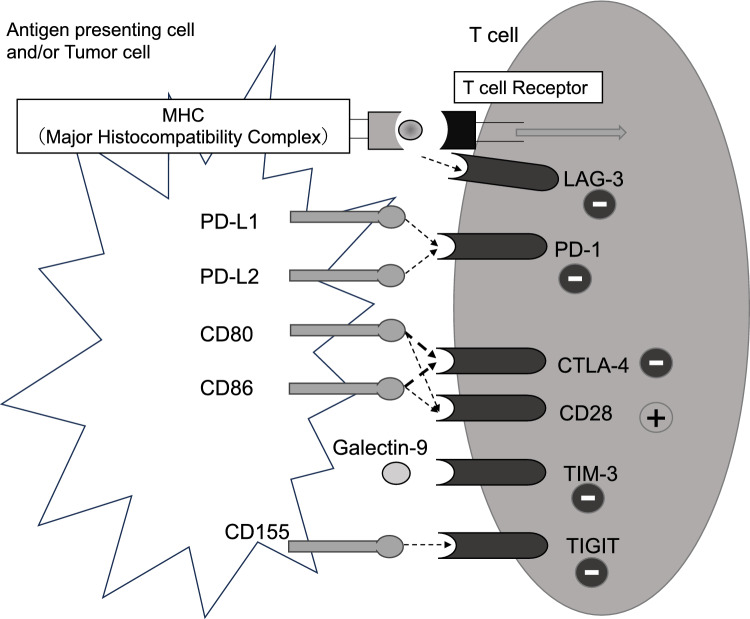
Co-stimulatory and inhibitory molecules regulate T cell activation and immune responses. Co-stimulatory molecules (e.g., CD28) promote T cell activation, proliferation, and cytokine production, whereas inhibitory molecules (e.g., CTLA-4, PD-1) suppress these processes

HPV is a small double-stranded DNA virus that is the major cause of cervical cancer [6]. Following the initial HPV infection, the viral DNA can integrate into the host genome [7] and this integration is linked to the progression from intraepithelial neoplasia to invasive carcinoma [8]. The E6 and E7 HPV oncoproteins promote cell proliferation and immortalization by inactivating cancer suppressor genes p53 and Rb [9]. In Cervical cancer tumor microenvironment complex interaction of immune cells that contribute to both tumor progression and immune evasion. CD8+ T cells play a key role in attacking tumor cells. They are stimulated by cytokines such as IL-2, which promotes their differentiation into cytotoxic T lymphocytes (CTLs), enhancing their ability to target and eliminate cancer cells. The cytotoxic activity of CTLs is mediated through molecules such as perforins and granzymes. Another important T cell population is CD4+ T cells, which help create an anti-tumor microenvironment by secreting inflammatory cytokines like IL-2. In contrast, regulatory T cells induced by immunosuppressive cytokines such as TGF-beta and IL-10, suppress immune response and contribute to immune evasion. Additionally, dendritic cells play a role in antigen presentation and the activation of immune cells. Tumor-associated macrophages, especially M2 macrophages, promote tumor cell growth and immune suppression [10]. Despite the detection of HPV-specific cytotoxic T-lymphocytes in tumors [11], the immune system often fails to eliminate the tumor, indicating that an immunosuppressive environment is present in cervical cancer [6]. This means that HPV shapes the microenvironment to mediate persistent infections that favor transformation and tumor development [10]. The antigens are recognized by T cells, and this recognition is controlled by costimulatory and inhibitory molecules. The cervical cancer cells overexpress an immune checkpoint molecule, PD-L1, which binds to the T cell immune checkpoint molecule PD-1 to suppress T cell activation. In addition, oncoproteins E6, E7 induce CTLA-4 expression and suppress T cell functions, thereby inducing an antitumor immune response. A key mechanism is the immune checkpoint pathway including PD-1, CTLA-4, and PD-L1, which increase immune resistance [12] (Fig. 1).

## Immune checkpoint antibodies in cervical cancer

The landscape of immune-checkpoint antibodies used in clinical trials for cervical cancer continues to expand. Representative agents include anti-PD-1 antibodies (pembrolizumab, cemiplimab, nivolumab, and balstilimab), anti-PD-L1 antibodies (durvalumab, atezolizumab), and anti-CTLA-4 antibodies (ipilimumab). These agents function by blocking checkpoint molecules, thereby reactivating the immune response against cancer cells.

### Immune checkpoint inhibitor monotherapy

#### Advanced and/or metastatic cervical cancer

Paclitaxel and platinum-based chemotherapy are now standard treatments for metastatic and recurrent cervical cancer [13]. In the GOG 240 trial, the addition of the anti-vascular endothelial growth factor (VEGF) antibody, bevacizumab, to chemotherapy increased survival [14]. However, the results revealed that options remained limited in term of second-line regimen for these patients; selected trials for patients with metastatic or recurrent cervical cancer are listed in Table 1. The KEYNOTE-028 trial (NCT02054806) [15] and KEYNOTE-158 trial (NCT02628067) [16] demonstrated the efficacy and the safety of pembrolizumab. The results of these trials led to FDA approval of pembrolizumab for cervical cancer with PD-L1 expressed tumors. The efficacy of monotherapy with nivolumab, another anti-PD-1 inhibitor for cervical cancer was investigated with NRG-GY002 (NCT02257528) [17] and CheckMate 358 trial (NCT02488759) [18].

**Table 1:** Selected trials for patients with metastatic or recurrent Cervical cancer

Author (year)	Trial name/ID	*P*	Treatment arms (dose)	Endpoints	Number of evaluable cases	Disease status	PD-L1 positive (CPS ≥ 1%) (%)	Prior CT (%) (except CCRT)	Prior RT (%)	ORR (%)	PFS (M)	OS (M)
Immune checkpoint inhibitor monotherapy												
Frenel et al. (2017) [15]	KEYNOTE-028/NCT02054806	Ib	Pembrolizumab 10 mg/kg Q2W	ORR	24	PD-L1-positive advanced solid tumors progressed after prior standard therapy	100 (modified proportion score)	100 [63% (2 or more lines)]	92	17	2	11
Lheureux et al. (2018) [19]	–/NCT01693783	II	Ipilimumab (Ipi)	safety/ORR	42	Metastatic cervical cancer with progression after at least 1 line of platinum chemotherapy	–	100 [50% (2 or more lines)]	83	2.9	2.5	8.5
Nauman et al. (2019) [18]	CheckMate 358/NCT02488759	I/II	Nivolumab (Nivo)(240 mg IV Q2W)	ORR	19 (cervical cancer cohort)	SCC Rec/meta disease with two or fewer prior systemic therapies	62.5 (cervical cancer cohort)	prior systemic therapy for metastatic disease (78.9%)	89.5	26.3	5.1	21.9
Nauman et al. (2019) [20]	CheckMate 358/NCT02488759	I/II	Nivo 3 mg/kg Q2W + Ipi 1 mg/kg Q6W (Combo A), Nivo 1 mg/kg + Ipi 3 mg/kg Q3W—>Nivo 240 mg Q2W (Combo B)	ORR	19 (without PST), 26 (with PST)	Rec/meta disease	–	–	–	32% (A), 46% (B) without PST	8.5 (B) without PST, 5.8 (B) with PST	NR (A/B) without PST, 10.3 (A), 25.4 (B) with PST
Chung et al. (2019) [16]	KEYNOTE-158/NCT02628067	II	Pembrolizumab 200 mg Q3W	ORR	98	Previously treated advanced cervical cancer	83.7	100	86.7	12.2 (0% PD-L1 negative)	2.1	9.4
Santin et al. (2020) [17]	NRG-GY002/NCT02257528	II	Nivolumab 3 mg/kg Q2W	Objective tumor response	25	Persistent/recurrent cervical cancer, failure of prior systemic therapy	72.7	100 (8.0% prior immunotherapy)	92.0	4	3.5	14.5
O'Malley et al. (2021) [21]	NCT02257528	II	Balstilimab (AGEN2034, anti-PD-1)	ORR	161	Previously treated advanced cervical cancer	61.5	100	–	15 ( 20% in PD-L1 positive)	–	–
Immune checkpoint inhibitor + chemotherapy												
Friedman et al. (2020) [22]	NCT02921269	II	Atezolizumab (1200 mg) + bevacizumab (15 mg/kg) Q3W	ORR	10	Rec/meta cervical cancer; progression after 1–2 prior therapies	–	100	73 (CCRT)	0	–	–
Zhang et al. (2022) [23]	ChiCTR 1900025992	II	SHR-1210 (Camrelizumab) + chemothearpy	ORR	35	Rec/meta without previous systemic chemotherapy	–	2.56 (adjuvant chemotherapy)	94.3	40.0	–	–
Colombo et al. (2021) [24]	KEYNOTE-826/NCT03635567	III	Chemotherapy ± Bevacizumab + Pembrolizumab or placebo	PFS/OS	617	Rec/meta without previous systemic chemotherapy	88.8	0	73.7 vs 71.5	–	10.4 vs 8.4	2-Year OS 50.4% vs 40.4%
Oakinin et al. (2023) [25]	BEATcc/NCT03556839	III	Chemotherapy + Bevacizumab + Atezolizumab or placebo	PFS	410	Rec/meta without previous systemic chemotherapy	–	0	68 vs 70	–	13.7 vs 10.4	32.1 vs 22.8
Immune checkpoint inhibitor combination												
O'Malley et al. (2021) [26]	NCT03495882	II	Balstilimab (AGEN2034) (3 mg/kg) Q2W + Zalifrelimab (AGEN 1884, anti-CTLA-4) (1 mg/kg) Q6W	ORR	155	Relapsed after a first-line, platinum-based treatment regimen	56.8	100	89.0	25.6	2.7	12.8
Gutierrez et al. (2023) [27]^a^	NCT03126110	I/II	INCAGN01876 + Nivolumab	TEAE/immune ORR	145	Previously Treated Advanced solid tumors	–	–	–	CR in 1 cervical cancer	–	–
Davis et al. (2022) [24]	NCT03241173	I/II	INCAGN01949 (anti-OX40) + Nivo + Ipi	TEAE/ORR	87	Rec/Meta solid tumors (including 2 cervical cancer patients)	–	–	–	Overall disease control rate 27.6%	–	–
Luke et al. (2023) [28]	NCT03219268	I	Tebotelimab monotherapy (PD-1- and LAG-3-targeting bispecific molecule) (Q2W)	Safety/MTD	269^b^	solid tumors or hematologic malignancies and disease progression on previous treatment	–	–	–	tumor decreases in 34% (59/172)	–	–
Immune checkpoint inhibitor + target therapy												
Lan et al. (2023) [29]^a^	CLAP/NCT03816553	II	SHR-1210 (Camrelizumab) IV (200 mg Q2W) + apatinib (tyrosine kinase inhibitor) (250 mg orally)	ORR	45	Rec/Meta with prior systemic chemotherapy	–	100	–	55.6	8.9	15.9
Immune checkpoint inhibitor + vaccination												
Bousquet et al (2023) [30]	NCT04405349	II	VB10.16 + Atezolizumab	AEs, ORR	50	HPV16 positive solid tumors	–	–	–	66.7% (disease control rate)	–	–
Lorusso et al. (2024) [31]^a^	NCT04646005	II	ISA101b + cemiplimab	ORR	113	Recurrent/metastatic HPV16 cervical cancer after first line chemotherapy	–	100	–	16.8	3.0	13.3

*P* phase, *CCRT* concurrent chemoradiation, *M* months, *Rec/Meta* recurrent/metastatic, *ORR* objective response rate, *PFS* progression free survival, *OS* overall survival, *NS* statistically not significant, *Q* quaque, *IV* intravenously, *RT* Radiotherapy, *MTD* maximum tolerated dose, *PST* prior systemic therapies, *CR* complete response

^a^ASCO meeting

^b^Including 17 cervical cancer patients

In contrast to the KEYNOTE-158 trial where pembrolizumab exclusively demonstrated responses in PD-L1-positive patients. A phase II study with the anti-PD-1 inhibitor balstilimab (NCT03104699) [21] showed efficacy in patients with PD-L1 negativity. Ipilimumab, a fully humanized monoclonal antibody targeting CTLA-4 that downregulates the T cell immune response, was investigated in a phase I/II study. Although ipilimumab was tolerable, this did not show significant single-agent activity [19]. The first phase III trial demonstrating beneficial overall survival (OS) of a checkpoint inhibitor in cervical cancer was EMPOWER-CERVICAL-1 (NCT03257267). This trial, involving the anti-PD-1 inhibitor cemiplimab as the second-line treatment [32] and enrolled patients who had progressed after platinum-containing therapy. Although median progression-free survival (PFS) was similar in both treatment groups (2.8 months for cemiplimab vs. 2.9 months for chemotherapy), the hazard ratio of 0.71 (95% CI 0.58–0.86; *P* < 0.001) indicated a significantly longer PFS conferred by cemiplimab than by chemotherapy. Notably, in this trial, patients with PD-L1 Combined Positive Score (CPS) < 1% demonstrated an objective response to cemiplimab, suggesting a potential effectiveness for PD-L1-negative patients [32]. The mechanism is not clear; however, immune cells within the tumor microenvironment are often suppressed by the PD-1 checkpoint molecule. Anti-PD-1 inhibitors may overcome this suppression, reactivating immune cells to exert anti-tumor effects. These trials demonstrated that monotherapy with anti-PD-1 drugs, such as cemiplimab, is effective for patients with recurrent cervical cancer including PD-L1-negative patients.

### Combination therapy

In combination therapy, the release of damage-associated molecular patterns (DAMPs) [33] from cells under the influence of coadministered cytotoxic drugs may potentially enhance the efficacy of ICIs by influencing the immune environment. A phase II trial combining atezolizumab with bevacizumab did not indicate efficacy when used as the second-line treatment for patients with cervical cancer who had previously received bevacizumab in chemotherapy [22]. The history of treatment with bevacizumab may influence the results.

#### Combination immunotherapy

Although monotherapy with ipilumumab, an anti-CTLA-4 inhibitor, did not demonstrate significant activity in cervical cancer, combination therapy with anti-PD-1 antibodies has shown potential effectiveness. The CheckMate 358 trial, a phase I/II multicohort trial with nivolumab and ipilumumab, demonstrated notable efficacy in patients treated with nivolumab and ipilumumab followed by that in patients who had undergone systemic therapies and were subsequently treated with nivolumab [20]. Another immune checkpoint doublet trial, with the anti-PD-1 inhibitor balstilimab and anti-CTLA-4 inhibitor zalifrelimab, also showed a high objective response rate (ORR) [26]. In combination therapy using an anti-T cell immunoreceptor with Ig and ITIM domains (TIGIT) inhibitor, tiragolumab and an anti-PD-L1 inhibitor, atezolizumab, the response rate was higher than with atezolizumab alone, although this did not reach statistical significance (SKYSCRAPER-04, NCT04300647) [34]. The combined treatment of pemblrolizumab, with another TIGIT inhibitor, vibostolimab, did not produce improvement in prognosis compared with pembrolizumab alone (KEYVIBE-005, NCT50007106) [35]. Other combinations, including the anti-GITR inhibitor (INCAGNO1876) with nivolumab, and/or ipilimumab, are currently being investigated (NCT03126110) [27]. Those trials indicate that combination immunotherapy may be a promising approach, although the selection of patients for the demonstration of efficacy requires further discussion.

#### Combination immunotherapy with chemotherapy

For a first-line treatment for advanced/metastatic cervical cancer, a phase III trial, KEYNOTE-826 trial (NCT03635567) demonstrated that combining immune checkpoint therapy using the anti-PD-1 inhibitor pembrolizumab with chemotherapy improved the prognosis. Bevacizumab was used in > 60% of patients in both arms of the trial (hazard ratio, 0.65; 95% CI 0.53–0.79; *P* < 0.001) [24]. The combination of chemotherapy with pembrolizumab showed improved survival with tolerable side effects, leading to FDA approval for the ICI combination treatment for patients with advanced/metastatic cervical cancer. Another notable phase III trial that assessed first-line combination therapy for metastatic or recurrent cervical cancer is BEATcc (NCT03556839), which indicated a benefit of adding atezolizumab, anti-PD-L1 inhibitor, to the GOG-240 trial regimen (chemotherapy with bevacizumab) [14] in patients with metastatic/recurrent cervical cancer [25]. The anti-VEGF antibody bevacizumab interferes with the composition and function of several immune cells within the tumor microenvironment, including T cells [36]. In a phase II trial conducted as a second-line treatment, no therapeutic effect was observed with the use of atezolizumab with bevacizumab [22]. The previous treatment history of patients with bevacizumab may have influenced the outcome. The BEATcc trial showed that the combination of bevacizumab and ICI may more effectively modulate the tumor microenvironment.

Another approach involves using combinations with drugs targeting checkpoints beyond PD-1, PD-L1, CTLA-4, or TIGIT. Co-stimulation molecules, including LAG-3, VISTA, ICOS, and OX40, serve as targets for combination drugs in patients with cervical cancer [37] (NCT05864144, NCT03829501). Selected trials for ongoing trials for patients with cervical cancer are listed in Table 2.

**Table 2:** Selected on-going trials for patients with cervical cancer

Trial name/ID	*P*	Treatment arms (dose)	Primary endpoints	Secondary endpoints	Planned number of patients	Disease status
1st line; immunotherapy + chemotherapy						
ENGOT-cx13/FERMATA/NCT03912415	III	BCD-100 (anti-PD-1) (3 mg/kg Q3W) + chemotherapy ± Bevacizumab vs placebo + chemotherapy ± Bevacizumab	OS	PFS, ORR, Disease control rate, Time to response, duration of response	316	Rec/Meta cervical cancer with no prior systemic treatment
1st line; immunotherapy + CCRT						
ATEZOLACC/NCT03612791	II	CCRT vs pre atezolizumab (1200 mg IV Q3W) + concurrent atezolizumab with CCRT + maintenance atezolizumab 20 cycles	PFS	-	189	FIGO 2009 stages IB1-IIA with positive pelvic nodal status, stage IIB-IVA, stage IVB with metastases limited to the paraaortic lymph nodes
NCT05492123	II	Nivolumab + Ipilimumab 200 mg + CCRT	3-Year PFS	3-year OS, ORR, Response duration	112	FIGO Stage IB2-IB3 node positive or Stage IIB-IVA
2nd or more line; immunotherapy						
NCT04380805	II	Cadonilimab (anti PD-1/CTLA-4 bispecific antibody)	ORR	PFS, Response duration	30	SCC or adenosquamous carcinoma, 1 or 2 prior chemotherapy regimen for recurrent or advanced disease
Immunotherapy combination						
NCT05864144	I/II	SNS-101 (anti-VISTA) ± Cemiplimab	ORR, AEs	Determine pharmacokinetic profile, PFS	129	Rec/Meta solid tumors
NCT03829501	II	Atezolizumab ± KY1044 (human anti-ICOS inhibitor)	AEs,SAEs, ORR	Best overall response, PFS, DOR	280	NSCLC, HNSCC, HCC, melanoma, cervical, esophageal, gastric, renal, pancreatic, triple negative breast cancer
2nd or more line; immunotherapy + targeting therapy						
NCT04483544	II	Pembrolizumab 200 mg IV Q3W + Olaparib (PARP inhibitor) 300 mg orally BID	Immune ORR	PFS, TEAEs, Duration of response	48	Rec/Meta progressed on 1st line chemotherapy
GOTIC-025/jRCT2031210096/NCT04641728*	II	Pembrolizumab 200 mg IV Q3W + Olaparib (PARP inhibitor) 300 mg orally BID	ORR	Immune ORR, PFS, Duration of response	28	Rec/Meta progressed after prior platinum based chemotherapy
NCT04652076	I/II	A; TC, B; NP137 (anti-Netrin-1) + TC, C; NP137 + Pembrolizumab, D; NP137 + Pembrolizumab + TC	DLT, ORR	Clinical Benefit Rate, PFS, OS	240	Rec/Meta endometrial cancer or cervix adeno- or epidermoid- carcinoma, with prior platinum based chemotherapy
NCT04865887	II	Pembrolizumab (200 mg IV Q3W) + Lenvatinib 20 mg orally	ORR	Duration of response, PFS/OS	35	Rec/Meta cervical cancer
Immunotherapy + vaccination						
NCT03444376	I/II	GX-188E (vaccine) + Pembrolizumab	DLT, ORR	DOR, PFS, OS	60	Red/Meta HPV-positive (HPV-16 or HPV-18) cervical cancer
NCT06099418	II	VB10.16 (vaccine) + Atezolizumab/placebo	ORR	DOR, PFS/OS	130	HPV16-positive, PD-L1-positive, Rec/Meta cervical cancer, refractory to pembrolizumab + chemotherapy ± bevacizumab

*P* phase, *M* months, *ORR* overall response rate, *PFS* progression free survival, *OS* overall survival, *Q* quaque, *Rec/Meta* recurrent/metastatic, *RT* radiation therapy, *SCC* squamous-cell carcinoma, *CCRT* concurrent chemoradiotherapy, *TEAE* Number of patient reporting treatment-emergent adverse events, *BID* twice daily, *NSCLC* non-small cell lung cancer, *HNSCC* head and neck squamous cell carcinoma, *HCC* hepatocellular carcinoma, *TC* paclitaxel + carboplatin

*ESMO (European Society for Medical Oncology) congress

#### Combination with targeted therapy

Targeting therapies have demonstrated a degree of effectiveness for cancer, and a synergistic effect is expected in combination with ICIs. For example, one approach involves combining ICIs with anti-angiogenic agents like bevacizumab. Tumor angiogenesis not only promotes tumor growth and metastasis but also creates an immunosuppressive microenvironment by reducing T-cell infiltration and increasing regulatory T cells and myeloid-derived suppressor cells (MDSCs). Anti-angiogenic therapy normalizes the tumor vasculature, facilitating T cell infiltration and enhancing the efficacy of ICIs [38]. The PD-1 inhibitor camrelizumab (SHR-1210) is efficacious in combination with chemotherapy as a first-line treatment and demonstrated a tolerable response with the tyrosine kinase inhibitor apatinib as a second-line treatment [23, 29]. Combining ICIs with PARP inhibitors is being explored for patients with recurrent cervical cancer [39], (GOTIC-025/NCT04641728). For uterine endometrial cancer, the combination of lenvatinib, which helps reduce tumor-associated macrophages (TAMs) and activate CD8+ T cells and pembrolizumab increased the prognosis [40, 41], potentially overcoming VEGF-mediated immunosuppression. The combination has also been investigated in patients with advanced cervical cancer (NCT04865887). A phase 1/2 trial for pembrolizumab with the NETrin monoclonal antibody that can inhibit tumor growth in endometrial cancer [42] are being investigated in patients with gynecologic cancer (GYNET/NCT04652076). Another strategy leverages targeted agents that modulate specific signaling pathways, such as inhibitors of VEGF, EGFR, or PI3K/AKT/mTOR. mTOR pathway inhibitors have been shown to reduce regulatory T cell activity and promote cytotoxic T cell responses. These effects may synergize with ICIs to improve anti-tumor immune activity [43]. mTORC1/2 inhibitor, onatasertib, and anti-PD-1 inhibitor, toripalimab demonstrated encouraging an ORR of 53.3% in 31 patients with cervical cancer (NCT04337463) [44].

### Immunotherapy for locally advanced cervical cancer

Concurrent chemoradiotherapy (CCRT) is used as the standard treatment for locally advanced cervical cancer (LACC). CCRT with a weekly cisplatin regimen has demonstrated improved survival compared with radiotherapy alone [45, 46]. Notably, radiotherapy can enhance immune responses when combined with PD-1 ICIs in melanoma [47]. This observation prompted the initiation of clinical trials combining ICIs with the standard treatment of CCRT in LACC. Selected trials for patients with LACC are listed in Table 3. The phase I trial, GOG-9929, demonstrated the safety of adjuvant ipilimumab following CCRT [48], and a phase II trial indicated a similar safety profile for adjuvant or maintenance pembrolizumab with CCRT [49]. Additionally, the NRG GY017 study demonstrated the safety of atezolizumab with CCRT for high-risk LACC with lymph node metastasis [50]. Both studies affirmed the safety of adjuvant or concurrent ICIs with CCRT. In phase III trials of ICIs for treating LACC, the CALLA trial (NCT03830866), is a randomized phase III trial that adds the PD-L1 inhibitor durvalumab to CCRT for 770 patients with LACC. In the durvalumab-adding population (*n* = 385), the PD-L1 CPS ≥ 1% was 28.7%, and in the placebo-adding population (*n* = 385), the PD-L1 CPS ≥ 1% was 33.2%. In the intention-to-treat population, PFS did not significantly differ between the durvalumab plus CCRT group and the placebo plus CCRT group (HR 0.84; 95% CI 0.65–1.08; *P* = 0.17) [51]. The inclusion of para-aortic lymph node-positive patients and other factors may have influenced the results. In another trial, the ENGOT-cx11/GOG-3047/KEYNOTE-A18 study (NCT04221945) involving 1060 patients, pembrolizumab combined with CCRT (*n* = 529) demonstrated a significant improvement in PFS compared with that in the placebo combined with CCRT group (*n* = 531), with a 24-month PFS of 67.8% vs 57.3%, respectively (*P* = 0.0020) [52]. The differences in the results between CALLA trial and KEYNOTE-A18 may be influenced by several factors, KEYNOTE-A18 trial targeted a higher risk population, including criteria such as lymph node size or number, and also differed in sample size and ICIs. The finding from the KEYNOTE-A18 supported the FDA approval of pembrolizumab plus chemoradiotherapy in high-risk cervical cancer. Although the use of ICIs for LACC present a favorable treatment option, the appropriate timing to initiate ICIs and the biomarker for response remain unclear. In the phase I NiCOL trial (NCT03298893), which included 16 patients with LACC, the safety and tolerance of nivolumab with and following CCRT were investigated [53]. The results indicated that nivolumab with and following CCRT is tolerable, with an ORR of 93.8%. Patients with progression-free status demonstrated an active stromal immune infiltrate, and tumor-infiltrating CD3+ T cells were found in closer proximity to PD-L1+ tumor cells than in patients with progressive disease. This suggests a specific tumor microenvironment is related to the response to the treatment of CCRT and ICIs in LACC. Additionally, the induction chemotherapy before CCRT improved survival compared with CCRT alone in LACC (INTERLACE trial) [54].

**Table 3:** Selected trials for patients with locally advanced cervical cancer

Author (year)	Trial name/ID	*P*	Treatment arms (Dose)	Endpoints	Number of evaluable cases	Disease status	PD-L1 positive (CPS ≥ 1%) (%)	ORR (%)	PFS (M)	OS (M)
Immunotherapy after CCRT										
Da Silva et al. (2021) [45]	GOG-9929/NCT01711515	I	Adjuvant Ipilimumab IV Q3W following CCRT	Safety, Secondary PFS/OS	21	FIGO stages IB2/IIA with positive PALN or FIGO stages IIB/IIIB/IVA with positive pelvic- and/or PALN	–	–	12-Month PFS 81%	12-Month OS 90%
Combination with CCRT										
Mayadev et al. (2025) [50]	NRG-GY017/NCT03738228	I	Atezolizumab	Toxicity	30	Locally advanced, node-positive cervical cancer	30–83%	–	2-Year DFS 59–79%	–
Monk et al. (2023) [51]	CALLA/NCT03830866	III	Durvalumab Q4W + CCRT vs placebo + CCRT	PFS	770	FIGO 2009 stage IB2–IIB lymph node positive, stage ≥ III any lymph node status	96 vs 97 (TAP score)	83 vs 81	12-Month PFS 76·0% vs 73.3%	–
Lorusso et al.^a^ (2023) [52]	ENGOT-cx11/GOG-3047/KEYNOTE-A18/NCT04221945	III	Pembrolizumab or placebo Q3W + CCRT- > 15 cycles Pembrolizumab or placebo Q6W	PFS/OS	1060	FIGO 2014 stage IB2-IIB with node-positive disease or stage III-IVA	–	–	24-Months PFS of 67.8% vs 57.3%	–
Nakamura et al.^b^ (2023) [55]	GOTIC-018/JMA-IIA00425	I	Nivolumab + CCRT	Safety	30	FIGO 2009 stage IB–IVA	46.7% (TPS)	93.3–100	12-Month PFS 100%	–
Rodrigues et al. (2023) [53]	NiCOL/NCT03298893	I	Nivolumab (240 mg) Q2W + CCRT- > Maintenance Nivolumab (6 months)	DLT	21	Immunotherapy-naïve adult patients, FIGO 2018 stages IB3-IVA	–	93.8%	–	–
Neoadjuvant chemotherapy with immunotherapy										
Li et al. (2024) [56]	NACI/NCT04516616	II	Camrelizumab (anti-PD-1) + chemotherapy- > surgery or CCRT	ORR	85	FIGO 2018 stage IB3,IIA2, IIB/IIIC1r	42 (CPS ≥ 10)	98	–	–

*P* phase, *M* months, *ORR* overall response rate, *PFS* progression free survival, *OS* overall survival, *CCRT* concurrent chemoradiotherapy, *Q* quaque, *PALN* para aortic lymph nodes, *SCC* squamous-cell carcinoma *TAP* tumour area positivity, *DLT* dose-limiting toxicities

^a^ESMO (European Society for Medical Oncology) congress 2023

^b^ASCO congress 2023

#### Neoadjuvant immune therapy for locally advanced cervical cancer

Furthermore, safety and efficacy were demonstrated by neoadjuvant immunotherapy demonstrated for locally advanced mismatch repaired-deficient colon cancer (NCT03026140) [57]. These results led to several trials with induction ICIs before CCRT for LACC. The multicohort phase I study, GOTIC-018 trial, explored nivolumab as induction and coadministration with CCRT, followed by nivolumab maintenance therapy in a cohort of the study [55]. Results showed that the pre- and coadministration of nivolumab is tolerable and exhibits durable efficacy. The COLIBRI trial, a phase II trial, is evaluating the combination of nivolumab and ipilimumab before CCRT and as maintenance for patients with LACC [58]. A trial of neoadjuvant immunotherapy, camrelizuamb, an anti-PD-1 inhibitor, with chemotherapy for LACC has exhibited promising antitumor activity, including 19% complete response [56]. Several ongoing trials are investigating ICIs with CCRT (ATEZOLACC/NCT03612791, NCT05492123), and the timing of implementation and an appropriate immune checkpoint arm, whether using monotherapy or combinations, are currently under evaluation for patients with LACC; the neoadjuvant immune therapy may also be considered for these patients.

### Retreatment strategies post-ICI

The rechallenge using ICIs presents a new avenue for addressing the unmet needs of patients who have experienced disease progression after initial treatment with these inhibitors. However, no trial has demonstrated the efficacy of retrying ICIs for patients with cervical cancer. In melanoma and non-small cell lung cancer, several trials have demonstrated the feasibility of ICI rechallenge for patients who progressed after initial treatment (NCT03334617, NCT03526887, NCT02743819) [59–61]. Notably, a randomized phase II trial for patients with melanoma who failed PD-1 ICI (NCT03033576) demonstrated that the combination of nivolumab with ipilimumab was more favorable than nivolumab monotherapy [62].

For patients with cervical cancer who have not responded to initial ICIs, planned or ongoing clinical trials are investigating potential options. These include a phase II trial of cadonilimab (bispecific anti-PD-1/CTLA-4) (NCT05824494) and a trial of therapeutic vaccine with atezolizumab or placebo (NCT06099418). While the rechallenge of ICIs for these patients may be a viable option, the detection of biomarkers to identify appropriate patients becomes crucial.

These ongoing trials will provide valuable insights into the feasibility and efficacy of ICI rechallenge in cervical cancer, and the identification of biomarkers will contribute to refining patient selection for this approach. Addressing the challenges associated with disease progression after initial treatment with ICIs remains a significant focus in advancing the therapeutic options for patients with cervical cancer.

## Further perspectives of immune therapy in cervical cancer

Based on the results of previous clinical trials, ICIs have consistently demonstrated efficacy in treating cervical cancer. However, the current stage reveals that only a partial population of patients is effectively treated, highlighting the urgent need for appropriate biomarkers to identify individuals who are most likely to benefit from specific therapies. PD-L1 is one of the predictive biomarkers for ICI treatments in solid tumors, and could be assessed by immunohistochemical staining. In the KEYNOTE-158 trial, PD-L1-negative patients showed no response to treatment [16]. Moreover, PD-L1 expression differs significantly between adenocarcinoma and squamous cell carcinoma (SCC) (14% vs. 54%) [63].

Tumor mutational burden (TMB), defined as the total number of somatic mutations per coding region of a tumor genome, is another potential predictive biomarker for response to ICIs. In the KEYNOTE-158 analysis, 21% of cervical cancer patients exhibited high TMB status. Across a cohort of 805 patients, including cervical cancer cases, durable tumor responses to pembrolizumab were observed [64].

Additionally, the presence of tumor-infiltrating lymphocytes (TILs) appears to correlate with improved survival. A higher ratio of CD8+ TILs to CD4+ Tregs is associated with better survival rates in cervical cancer (5-year survival rate: 82% vs. 44%) [65]. While these biomarkers show promise for identifying appropriate patient groups for ICI therapy and predicting prognosis, significant challenges remain. Further research, incorporating real-world clinical outcomes, is essential to refine and validate these predictive tools.

The rechallenge of ICIs in cervical cancer remains unclear. Therefore, the next step involves exploring immune checkpoint combination therapy with other ICIs or alternative therapies such as targeting agents, immune therapies (including vaccination), or adoptive cell therapies. Addressing the treatment strategy for patients experiencing immune checkpoint failure represents an unmet need.

Bispecific molecules are also being explored as a novel type of ICIs. A phase I study with the PD-1 and LAG-3 targeting bispecific molecule, tebotelimab, demonstrated efficacy in a small cohort of patients with cervical cancer [28]. Another ongoing trial involves the bispecific agent anti-PD-1/CTLA-4, cadonilimab [66].

Trials with novel types of ICIs targeting mutations with high affinity are ongoing. BCD-100 (prolgolimab), an anti-PD-1 inhibitor that targets the Fc-silencing LALA mutation, has shown significant antitumor activity in melanoma [67]. The FERMATA/ENGOT-cx13 trial is an ongoing phase III trial exploring BCD-100 as a first-line treatment for advanced or recurrent cervical cancer [67]. At this moment, novel type of ICI have demonstrated limited effectiveness as monotherapies. Therefore, these novel types of ICIs are mainly being evaluated in combination with anti-PD-1 or PD-L1 inhibitors, aiming for a synergistic effect [68].

### Adoptive cell therapy (tumor-infiltrating lymphocytes and engineered TCR T cell-based therapy, CAR-T)

Adoptive cell transfer treatment using tumor-infiltrating lymphocytes (TILs) would be a favorable therapeutic option for solid tumors. The FDA approved the first TIL therapy for advanced melanoma in 2024. TILs are isolated from the patient’s tumor tissue and stimulated and expanded to large number by interleukin (IL)−2 treatment. IL-2 helps T cell proliferation and activation. TIL therapy can target cancer cells carrying the patient’s specific neoantigens [69]. TIL therapy may adapt to tumor heterogeneity because this contains T cells with multiple T cell receptor clones [70], making this a favorable treatment. Several trials using TIL therapy for cervical cancer have been reported, including one demonstrating that TILs for cervical cancer can target not only neoantigen but also cancer germline antigens. A single infusion of TILs selected for reactivity against HPV E6 and E7 showed tumor regression in patients with metastatic cervical cancer (NCT01585428) [71]. In a phase II study with LN-145, TIL therapy in patients with recurrent cervical cancer after chemotherapy demonstrated therapeutic efficacy with the ORR of 44% and the disease control rate of 89% (NCT03108495), with one patient showing a complete response [72]. While TIL therapy has shown promise as a treatment for melanoma, the presence of more than three lesions or metastasis to the liver and brain may be associated with treatment resistance [73]. Similarly, selecting the appropriate candidates may be crucial to achieve therapeutic efficacy in cervical cancer.

In recent years, therapy utilizing chimeric antigen receptor (CAR)-T cells, designed to recognize and target cancer-specific antigens, has shown success and gained approval from regulatory agencies such as the FDA for hematologic malignancies. However, the expression of cancer-specific antigens in solid tumors is limited. The first phase I/II clinical trial of mesothelin CAR-Ts targeting mesothelin-expressing tumors, including cervical and ovarian cancer (NCT01583686), was terminated because of slow accrual. The efficacy was low in this study, with only one of 15 patients showing stable disease for > 3.5 months [74]. And for solid tumors, CAR-T cell therapy carries the risk of serious on-target, off-tumor toxicity due to CAR-T cell-mediated cytotoxicity against non-malignant tissues expressing the target antigen [75].

In the first phase I/II trial of MAGE-A3 TCR-T (NCT02111850) to treat cervical cancer, a complete response was observed for > 29 months in one patient. The oncoproteins HPV-E6 and HPV-E7 are viral antigens expressed in cancers but not in healthy tissues. Therefore, they are attractive targets for genetically engineered T-cell therapy in HPV-related epithelial cancers [76]. A phase I/II trial (NCT02280811) with TCR-T targeting HPV-E6 for HPV16–positive patients, including six patients with cervical cancer, showed objective responses in two patients [77]. In another phase I trial (NCT02858310), TCR-engineered T cells targeting E7 for patients with metastatic HPV-associated epithelial cancers, including 5 patients with cervical cancer (2 of whom had been treated with ICIs), demonstrated objective clinical responses in 6 out of 12 patients [78]. These trials demonstrate an alternative treatment strategy for patients with cervical cancer, even in cases of progression after ICIs. While CAR-T cells target surface antigens, engineered TCR T cells can target intracellular antigens, but their limitation lies in HLA restrictions. Currently, HLA A*02:01 is a major target for engineered TCR T cell trials, although the distribution of HLA varies by region. The efficacy of engineered T cells for cervical cancers is currently limited and supported by clinical data, warranting further studies.

### Therapeutic cancer vaccination in cervical cancer

Cancer vaccination therapy aims to stimulate the immune system by introducing tumor antigens and inducing or amplifying an antitumor immune response. The combination with ICIs with vaccination may enhance sensitivity by overcoming tumor immune resistance. Several types of tumor-specific vaccines are used. For example, peptide vaccines target specific protein fragments while DNA vaccines deliver genetic particles to produce antigens and viral vector vaccines by using modified viruses to introduce antigens. Those antigens are loaded to antigen presenting cells and stimulate T cell-based responses [79]. In the KEYNOTE-567 trial, the combination with DNA vaccination with pembrolizumab showed a better response in patients with PD-L1-positive, HPV-16, and squamous cell carcinoma than pembrolizumab alone (NCT03444376) [80]. In another trial, a HPV16 therapeutic cancer vaccine with atezolizumab showed a favorable response in PD-L1-positive patients. HPV16-circulating tumor DNA levels may predict tumor response (NCT04405349) [30]. A trial for patients with cervical cancer who relapsed after pembrolizumab is testing the efficacy of this vaccine and atezolizumab or placebo (NCT06099418). The combination of peptide vaccine, ISA101b vaccine and cemiplimab demonstrated clinical benefit especially in such patients with high PD-L1 expression [31]. Therefore, cancer vaccination therapy can be effective in appropriate patients. However, a challenge remains that the immune response does not persist and diminishes over time. These emerging therapies represent promising directions for advancing treatment options for patients with cervical cancer and warrant further exploration in clinical trials.

## Conclusions

Immune-related therapies have ushered in a new era for treating cervical cancer, specifically addressing unmet clinical needs in advanced or metastatic cervical cancer. Despite notable progress, challenges persist, encompassing unresponsive tumors to immune therapies, issues related to immune therapy resistance, and the emergence of immune-related adverse events. To fully capitalize on the effectiveness of immunotherapy, extensive research efforts must be maintained from basic studies to clinical trials. This comprehensive approach will help identify suitable treatment regimens that incorporate immunotherapy for specific patient populations. Continuous research and development will play a pivotal role in refining and expanding the application of immunotherapeutic strategies, ultimately improving outcomes for individuals affected by cervical cancer.

## References

[1] Sung H, Ferlay J, Siegel RL et al (2021) Global cancer statistics 2020: GLOBOCAN estimates of incidence and mortality worldwide for 36 cancers in 185 countries. CA Cancer J Clin 71:209–249. 10.3322/caac.21660 33538338 10.3322/caac.21660

[2] FUTURE II Study Group (2007) Quadrivalent vaccine against human papillomavirus to prevent high-grade cervical lesions. N Engl J Med 356:1915–1927. 10.1056/NEJMoa061741 17494925 10.1056/NEJMoa061741

[3] Francoeur AA, Liao C-I, Caesar MA et al (2022) The increasing incidence of stage IV cervical cancer in the USA: what factors are related? Int J Gynecol Cancer. 10.1136/ijgc-2022-003728 35981903 10.1136/ijgc-2022-003728

[4] Goodwin EC, DiMaio D (2000) Repression of human papillomavirus oncogenes in HeLa cervical carcinoma cells causes the orderly reactivation of dormant tumor suppressor pathways. Proc Natl Acad Sci 97:12513–12518. 10.1073/pnas.97.23.12513 11070078 10.1073/pnas.97.23.12513 PMC18795

[5] Walsh RJ, Tan DSP (2021) The role of immunotherapy in the treatment of advanced cervical cancer: Current status and future perspectives. J Clin Med 10(19):4523. 10.3390/jcm10194523. 10.3390/jcm10194523 PMC8509251 34640541

[6] Piersma SJ (2011) Immunosuppressive tumor microenvironment in cervical cancer patients. Cancer Microenviron 4:361–375. 10.1007/s12307-011-0066-7. 21626415 10.1007/s12307-011-0066-7 PMC3234326

[7] Wentzensen N, Vinokurova S, Von M et al (2004) Systematic review of genomic integration sites of human papillomavirus genomes in epithelial dysplasia and invasive cancer of the female lower genital tract. Cancer Res 64(11):3878-84. 10.1158/0008-5472.CAN-04-0009. 15172997 10.1158/0008-5472.CAN-04-0009

[8] Hopman AHN, Smedts F, Dignef W et al (2004) Transition of high-grade cervical intraepithelial neoplasia to micro-invasive carcinoma is characterized by integration of HPV 16/18 and numerical chromosome abnormalities. J Pathol 202:23–33. 10.1002/path.1490 14694518 10.1002/path.1490

[9] Werness BA, Levine AJ, Howley PM (1990) Association of human papillomavirus types 16 and 18 E6 proteins with p53. Science 248:76–79. 10.1126/science.2157286 2157286 10.1126/science.2157286

[10] Trujillo-Cirilo L, Weiss-Steider B, Vargas-Angeles CA et al (2023) Immune microenvironment of cervical cancer and the role of IL-2 in tumor promotion. Cytokine 170:156334. 10.1126/10.1016/j.cyto.2023.156334. 37598478 10.1016/j.cyto.2023.156334

[11] Evans EM, Man S, Evans AS et al (1997) Infiltration of cervical cancer tissue with human papillomavirus-specific cytotoxic T-lymphocytes. Cancer Res 57:2943–2950 9230206

[12] Pardoll DM (2012) The blockade of immune checkpoints in cancer immunotherapy. Nat Rev Cancer 12:252–264 22437870 10.1038/nrc3239 PMC4856023

[13] Moore DH, Blessing JA, McQuellon RP et al (2004) Phase III study of cisplatin with or without paclitaxel in stage IVB, recurrent, or persistent squamous cell carcinoma of the cervix: A Gynecologic Oncology Group study. J Clin Oncol 22:3113–3119. 10.1200/JCO.2004.04.170 15284262 10.1200/JCO.2004.04.170

[14] Tewari KS, Sill MW, Long HJ et al (2014) Improved survival with bevacizumab in advanced cervical cancer. N Engl J Med 370:734–743. 10.1056/NEJMoa1309748 24552320 10.1056/NEJMoa1309748 PMC4010094

[15] Frenel J-S, Le TourneauO’neil CB et al (2017) Safety and efficacy of pembrolizumab in advanced, programmed death ligand 1-positive cervical cancer: results from the phase Ib KEYNOTE-028 trial. J Clin Oncol 35:4035–4041. 10.1200/JCO 29095678 10.1200/JCO.2017.74.5471

[16] Chung HC, Ros W, Delord J-P et al (2019) Efficacy and safety of pembrolizumab in previously treated advanced cervical cancer: results from the phase II KEYNOTE-158 study. J Clin Oncol 37(17):1470–1478. 10.1200/JCO.18.01265 30943124 10.1200/JCO.18.01265

[17] Santin AD, Deng W, Frumovitz M et al (2020) Phase II evaluation of nivolumab in the treatment of persistent or recurrent cervical cancer (NCT02257528/NRG-GY002). Gynecol Oncol 157:161–166. 10.1016/j.ygyno.2019.12.034 31924334 10.1016/j.ygyno.2019.12.034 PMC7127981

[18] Naumann W, Hollebecque A, Meyer T, et al (2019) Safety and efficacy of nivolumab monotherapy in recurrent or metastatic cervical, vaginal, or vulvar carcinoma: results from the phase I/II CheckMate 358 trial. J Clin Oncol 1;37(31):2825–2834. 10.1200/JCO.19.00739. 31487218 10.1200/JCO.19.00739 PMC6823884

[19] Lheureux S, Butler MO, Clarke B et al (2018) Association of ipilimumab with safety and antitumor activity inwomen with metastatic or recurrent human papillomavirus-related cervical carcinoma. JAMA Oncol. 10.1001/jamaoncol.2017.3776 29145543 10.1001/jamaoncol.2017.3776 PMC6145732

[20] Naumann RW, Oaknin A, Meyer T et al (2019) Efficacy and safety of nivolumab (Nivo) + ipilimumab (Ipi) in patients (pts) with recurrent/metastatic (R/M) cervical cancer: Results from CheckMate 358. Ann Oncol 30:v898–v899. 10.1093/annonc/mdz394.059

[21] O’Malley DM, Oaknin A, Monk BJ et al (2021) Phase II study of the safety and efficacy of the anti-PD-1 antibody balstilimab in patients with recurrent and/or metastatic cervical cancer. Gynecol Oncol 163:274–280. 10.1016/j.ygyno.2021.08.018 34452745 10.1016/j.ygyno.2021.08.018

[22] Friedman CF, Snyder Charen A, Zhou Q et al (2020) Phase II study of atezolizumab in combination with bevacizumab in patients with advanced cervical cancer. J Immunother Cancer. 10.1136/jitc-2020-001126 33004542 10.1136/jitc-2020-001126 PMC7534695

[23] Zhang X, Chen J, Liu N et al (2022) Camrelizumab (SHR-1210) with carboplatin and albumin-binding paclitaxel in patients with metastatic or recurrent cervical cancer: an open-label, phase 2 trial. J Cancer Res Ther 18:482–487. 10.4103/jcrt.jcrt_1851_21 35645118 10.4103/jcrt.jcrt_1851_21

[24] Colombo N, Dubot C, Lorusso D et al (2021) Pembrolizumab for persistent, recurrent, or metastatic cervical cancer. N Engl J Med 385:1856–1867. 10.1056/nejmoa2112435 34534429 10.1056/NEJMoa2112435

[25] Oaknin A, Gladieff L, Martínez-García J et al (2023) Atezolizumab plus bevacizumab and chemotherapy for metastatic, persistent, or recurrent cervical cancer (BEATcc): a randomised, open-label, phase 3 trial. Lancet. 10.1016/S0140-6736(23)02405-4 38048793 10.1016/S0140-6736(23)02405-4

[26] Omalley DM, Neffa M, Monk BJ et al (2021) Dual PD-1 and CTLA-4 checkpoint blockade using balstilimab and zalifrelimab combination as second-line treatment for advanced cervical cancer: an open-label phase II study. J Clin Oncol 40:762–771. 10.1200/JCO.21 34932394 10.1200/JCO.21.02067 PMC8887945

[27] Gutierrez M, Sarkis Balmanoukian A, Shields AF, et al (2023) 2541 poster session A phase 1/2 study of the safety, tolerability, and preliminary efficacy of the anti-GITR monoclonal antibody, INCAGN01876, combined with immunotherapies (IO) in patients (Pts) with advanced cancers. J Clin Oncol. 41, Number 16_suppl. 10.1200/JCO.2023.41.16_suppl.2541

[28] Luke JJ, Patel MR, Blumenschein GR et al (2023) The PD-1- and LAG-3-targeting bispecific molecule tebotelimab in solid tumors and hematologic cancers: a phase 1 trial. Nat Med. 10.1038/s41591-023-02593-0 37857711 10.1038/s41591-023-02593-0 PMC10667103

[29] Lan C, Lu H, Liu J et al (2023) Long-term survival outcomes and immune checkpoint inhibitor (ICI) retreatment in patients with advanced cervical cancer who received camrelizumab plus apatinib in the CLAP study. J Clin Oncol 41:e17513–e17513. 10.1200/JCO.2023.41.16_suppl.e17513 10.1002/cac2.12547 PMC11194449 38741375

[30] Bousquet PA, Berg KC, Blaga M et al (2023) 667 Predictive value of circulating tumor DNA in patients with advanced HPV16-positive cervical cancer treated with VB10.16 in combination with atezolizumab. In: Regular and Young Investigator Award abstracts. BMJ Publishing Group Ltd, p A757

[31] Lorusso D, Oaknin A, Santos Borges G et al (2024) Combination of cemiplimab and ISA101b vaccine for the treatment of recurrent/metastatic HPV16 cervical cancer. J Clin Oncol 42:5522–5522. 10.1200/JCO.2024.42.16_suppl.5522

[32] Tewari KS, Monk BJ, Vergote I et al (2022) Survival with cemiplimab in recurrent cervical cancer. N Engl J Med 386:544–555. 10.1056/NEJMoa2112187 35139273 10.1056/NEJMoa2112187

[33] Hangai S, Ao T, Kimura Y et al (2016) PGE2 induced in and released by dying cells functions as an inhibitory DAMP. Proc Natl Acad Sci USA 113:3844–3849. 10.1073/pnas.1602023113 27001836 10.1073/pnas.1602023113 PMC4833254

[34] Salani R, McCormack M, Kim Y-M et al (2024) A non-comparative, randomized, phase II trial of atezolizumab or atezolizumab plus tiragolumab for programmed death-ligand 1-positive recurrent cervical cancer (SKYSCRAPER-04). Int J Gynecol Cancer. 10.1136/ijgc-2024-005588 38858106 10.1136/ijgc-2024-005588

[35] Le Tourneau C, Rojas CI, Leary A et al (2024) 22O Association of biomarkers with response to coformulated vibostolimab/pembrolizumab (vibo/pembro) in metastatic cervical cancer (CC): Exploratory analysis from the phase II KEYVIBE-005 study. ESMO Open 9:103522. 10.1016/j.esmoop.2024.103522

[36] De Aguiar RB, De Moraes JZ (2019) Exploring the immunological mechanisms underlying the anti-vascular endothelial growth factor activity in tumors. Front Immunol 10:1023. 10.3389/fimmu.2019.01023. 10.3389/fimmu.2019.01023 PMC6530399 31156623

[37] Davis AA, Patel VG (2019) The role of PD-L1 expression as a predictive biomarker: an analysis of all US food and drug administration (FDA) approvals of immune checkpoint inhibitors. J Immunother Cancer. 10.1186/s40425-019-0768-9 31655605 10.1186/s40425-019-0768-9 PMC6815032

[38] Lee WS, Yang H, Chon HJ et al (2020) Combination of anti-angiogenic therapy and immune checkpoint blockade normalizes vascular-immune crosstalk to potentiate cancer immunity. Exp Mol Med 52:1475–1485. 10.1038/s12276-020-00500-y 32913278 10.1038/s12276-020-00500-y PMC8080646

[39] Diaz JP, Duan W, Schroeder E et al (2021) Immunotherapy in combination with PARP inhibition in advanced cervical cancer patients functionally competent or deficient for the Fanconi anemia repair pathway. J Clin Oncol 39:TPS5597. 10.1200/JCO.2021.39.15_suppl.TPS5597

[40] Makker V, Colombo N, Casado Herráez A et al (2022) Lenvatinib plus pembrolizumab for advanced endometrial cancer. N Engl J Med 386:437–448. 10.1056/nejmoa2108330 35045221 10.1056/NEJMoa2108330 PMC11651366

[41] Kato Y, Tabata K, Kimura T et al (2019) Lenvatinib plus anti-PD-1 antibody combination treatment activates CD8+ T cells through reduction of tumor-associated macrophage and activation of the interferon pathway. PLoS ONE. 10.1371/journal.pone.0212513 30811474 10.1371/journal.pone.0212513 PMC6392299

[42] Cassier PA, Navaridas R, Bellina M et al (2023) Netrin-1 blockade inhibits tumour growth and EMT features in endometrial cancer. Nature 620:409–416. 10.1038/s41586-023-06367-z 37532934 10.1038/s41586-023-06367-z PMC10412451

[43] Powell JD, Pollizzi KN, Heikamp EB et al (2012) Regulation of immune responses by mTOR. Annu Rev Immunol 30:39–68. 10.1146/annurev-immunol-020711-075024 22136167 10.1146/annurev-immunol-020711-075024 PMC3616892

[44] Yuan L, Shu P, Li X et al (2024) A phase 1/2 study of the TORC1/2 inhibitor onatasertib combined with toripalimab in patients with advanced solid tumors: cervical cancer cohort. J Clin Oncol. 42, Number 16_suppl. 10.1200/JCO.2024.42.16_suppl.5509

[45] Rose PG, Bundy BN, Watkins EB et al (1999) Concurrent cisplatin-based radiotherapy and chemotherapy for locally advanced cervical cancer. N Engl J Med 340:1144–1153. 10.1056/NEJM199904153401502 10202165 10.1056/NEJM199904153401502

[46] Morris M, Eifel PJ, Burke TW et al (1995) Treatment of locally advanced cervical cancer with concurrent radiation and intra-arterial chemotherapy. Gynecol Oncol 57:72–78 7705704 10.1006/gyno.1995.1101

[47] Sharabi AB, Nirschl CJ, Kochel CM et al (2015) Stereotactic radiation therapy augments antigen-specific PD-1-mediated antitumor immune responses via cross-presentation of tumor antigen. Cancer Immunol Res 3:345–355. 10.1158/2326-6066.CIR-14-0196 25527358 10.1158/2326-6066.CIR-14-0196 PMC4390444

[48] da Silva DM, Enserro DM, Mayadev JS et al (2021) Immune activation in patients with locally advanced cervical cancer treated with ipilimumab following definitive chemoradiation (GOG-9929). Clin Cancer Res 26:5621–5630. 10.1158/1078-0432.CCR-20-0776 10.1158/1078-0432.CCR-20-0776 PMC7642021 32816895

[49] Duska LR, Scalici JM, Temkin SM et al (2020) Results of an early safety analysis of a study of the combination of pembrolizumab and pelvic chemoradiation in locally advanced cervical cancer. Cancer 126:4948–4956. 10.1002/cncr.33136 32910478 10.1002/cncr.33136

[50] Mayadev J, Zamarin D, Deng W et al (2025) Neoadjuvant or concurrent atezolizumab with chemoradiation for locally advanced cervical cancer: a randomized phase I trial. Nat Commun. 16(1):553. 10.1038/s41467-024-55200-2. 39788967 10.1038/s41467-024-55200-2 PMC11718273

[51] Monk BJ, Toita T, Wu X et al (2023) Durvalumab versus placebo with chemoradiotherapy for locally advanced cervical cancer (CALLA): a randomised, double-blind, phase 3 trial. Lancet Oncol . 24(12):1334–1348. 10.1016/S1470-2045(23)00479-5. 10.1016/S1470-2045(23)00479-5 38039991

[52] Lorusso D, Xiang Y, Hasegawa K et al (2023) Pembrolizumab plus chemoradiotherapy for high-risk locally advanced cervical cancer: a randomized, double-blind, phase III ENGOT-cx11/GOG-3047/KEYNOTE-A18 study. Ann Oncol 34:S1279–S1280. 10.1016/j.annonc.2023.10.032

[53] Rodrigues M, Vanoni G, Loap P et al (2023) Nivolumab plus chemoradiotherapy in locally-advanced cervical cancer: the NICOL phase 1 trial. Nat Commun. 10.1038/s41467-023-39383-8 37349318 10.1038/s41467-023-39383-8 PMC10287640

[54] McCormack M, Gallardo Rincón D, Eminowicz G et al (2023) LBA8 a randomised phase III trial of induction chemotherapy followed by chemoradiation compared with chemoradiation alone in locally advanced cervical cancer: the GCIG INTERLACE trial. Ann Oncol 34:S1276. 10.1016/j.annonc.2023.10.028

[55] Nakamura K, Yabuno A, Satoh T et al (2023) Efficacy and final safety analysis of pre- and co-administration of nivolumab (Nivo) with concurrent chemoradiation (CCRT) followed by Nivo maintenance therapy in patients (pts) with locally advanced cervical carcinoma (LACvCa): Results from the phase I trial, GOTIC-018. J Clin Oncol 41:5519–5519. 10.1200/JCO.2023.41.16_suppl.5519

[56] Li K, Chen J, Hu Y et al (2024) Neoadjuvant chemotherapy plus camrelizumab for locally advanced cervical cancer (NACI study): a multicentre, single-arm, phase 2 trial. Lancet Oncol 25:76–85. 10.1016/S1470-2045(23)00531-4 38048802 10.1016/S1470-2045(23)00531-4

[57] Chalabi M, Verschoor YL, Tan PB et al (2024) Neoadjuvant immunotherapy in locally advanced mismatch repair-deficient colon cancer. N Engl J Med 390:1949–1958. 10.1056/NEJMoa2400634 38838311 10.1056/NEJMoa2400634

[58] Ray-Coquard IL, Kaminsky-Forrett M-C, Ohkuma R et al (2023) In situ immune impact of nivolumab + ipilimumab combination before standard chemoradiation therapy (RTCT) for FIGO IB3-IVA in patients (pts) with cervical squamous carcinoma: COLIBRI trial, a GINECO study. J Clin Oncol 41:5501–5501. 10.1200/JCO.2023.41.16_suppl.550137847874

[59] Besse B, Awad M, Forde P et al (2021) OA07.08 HUDSON: an open-label, multi-drug, biomarker-directed, phase II platform study in patients with NSCLC, who progressed on anti-PD(L)1 therapy. J Thorac Oncol 16:S118–S119. 10.1016/j.jtho.2021.01.299

[60] Olson DJ, Eroglu Z, Brockstein B et al (2021) Pembrolizumab plus ipilimumab following anti-PD-1/L1 failure in melanoma. J Clin Oncol 39:2647–2655. 10.1200/JCO.21.00079 33945288 10.1200/JCO.21.00079 PMC8376314

[61] Ponce Aix S, Carcereny Costa E, Bosch-Barrera J et al (2021) 160P Pembrolizumab re-challenge in patients with relapsed non-small cell lung cancer (NSCLC): a preliminary report of the REPLAY phase II trial—cohort I. Ann Oncol 32:S1450. 10.1016/j.annonc.2021.10.179

[62] VanderWalde A, Bellasea SL, Kendra KL et al (2023) Ipilimumab with or without nivolumab in PD-1 or PD-L1 blockade refractory metastatic melanoma: a randomized phase 2 trial. Nat Med 29:2278–2285. 10.1038/s41591-023-02498-y 37592104 10.1038/s41591-023-02498-y PMC10708907

[63] Heeren AM, Punt S, Bleeker MC et al (2016) Prognostic effect of different PD-L1 expression patterns in squamous cell carcinoma and adenocarcinoma of the cervix. Mod Pathol 29:753–763. 10.1038/modpathol.2016.64 27056074 10.1038/modpathol.2016.64 PMC4931542

[64] Marabelle A, Fakih M, Lopez J et al (2020) Association of tumour mutational burden with outcomes in patients with advanced solid tumours treated with pembrolizumab: prospective biomarker analysis of the multicohort, open-label, phase 2 KEYNOTE-158 study. Lancet Oncol 21:1353–1365. 10.1016/S1470-2045(20)30445-9 32919526 10.1016/S1470-2045(20)30445-9

[65] Shah W, Yan X, Jing L et al (2011) A reversed CD4/CD8 ratio of tumor-infiltrating lymphocytes and a high percentage of CD4(+)FOXP3(+) regulatory T cells are significantly associated with clinical outcome in squamous cell carcinoma of the cervix. Cell Mol Immunol 8:59–66. 10.1038/cmi.2010.56 21200385 10.1038/cmi.2010.56 PMC4002991

[66] Wu X, Ji J, Lou H et al (2022) Efficacy and safety of cadonilimab, an anti-PD-1/CTLA4 bi-specific antibody, in previously treated recurrent or metastatic (R/M) cervical cancer: a multicenter, open-label, single-arm, phase II trial (075). Gynecol Oncol 166:S47–S48. 10.1016/S0090-8258(22)01293-8

[67] Tjulandin S, Demidov L, Moiseyenko V et al (2021) Novel PD-1 inhibitor prolgolimab: expanding non-resectable/metastatic melanoma therapy choice. Eur J Cancer 149:222–232. 10.1016/j.ejca.2021.02.030 33872982 10.1016/j.ejca.2021.02.030

[68] Borgeaud M, Sandoval J, Obeid M, et al (2023) Novel targets for immune-checkpoint inhibition in cancer. Cancer Treat Rev 120:102614. 10.1016/j.ctrv.2023.102614 10.1016/j.ctrv.2023.102614 37603905

[69] Mullard A (2024) FDA approves first tumour-infiltrating lymphocyte (TIL) therapy, bolstering hopes for cell therapies in solid cancers. Nat Rev Drug Discov 23:238–238. 10.1038/d41573-024-00035-1 38374249 10.1038/d41573-024-00035-1

[70] Wang S, Sun J, Chen K, et al (2021) Perspectives of tumor-infiltrating lymphocyte treatment in solid tumors. BMC Med 19(1):140. 10.1186/s12916-021-02006-4 10.1186/s12916-021-02006-4 PMC8194199 34112147

[71] Stevanović S, Draper LM, Langhan MM et al (2015) Complete regression of metastatic cervical cancer after treatment with human papillomavirus-targeted tumor-infiltrating T cells. J Clin Oncol 33:1543–1550. 10.1200/JCO.2014.58.9093 25823737 10.1200/JCO.2014.58.9093 PMC4417725

[72] Jazaeri AA, Zsiros E, Amaria RN et al (2019) Safety and efficacy of adoptive cell transfer using autologous tumor infiltrating lymphocytes (LN-145) for treatment of recurrent, metastatic, or persistent cervical carcinoma. J Clin Oncol 37:2538–2538. 10.1200/JCO.2019.37.15_suppl.2538

[73] Chesney J, Lewis KD, Kluger H et al (2022) Efficacy and safety of lifileucel, a one-time autologous tumor-infiltrating lymphocyte (TIL) cell therapy, in patients with advanced melanoma after progression on immune checkpoint inhibitors and targeted therapies: Pooled analysis of consecutive cohorts of the C-144-01 study. J Immunother Cancer. 10.1136/jitc-2022-005755 36600653 10.1136/jitc-2022-005755 PMC9748991

[74] Castelletti L, Yeo D, van Zandwijk N, Rasko JEJ (2021) Anti-Mesothelin CAR T cell therapy for malignant mesothelioma. Biomark Res 9(1):11. 10.1186/s40364-021-00264-1. 33588928 10.1186/s40364-021-00264-1 PMC7885509

[75] Flugel CL, Majzner RG, Krenciute G et al (2023) Overcoming on-target, off-tumour toxicity of CAR T cell therapy for solid tumours. Nat Rev Clin Oncol 20:49–62. 10.1038/s41571-022-00704-3 36418477 10.1038/s41571-022-00704-3 PMC10278599

[76] Hinrichs CS (2016) Molecular pathways: Breaking the epithelial cancer barrier for chimeric antigen receptor and t-cell receptor gene therapy. Clin Cancer Res 22:1559–1564. 10.1158/1078-0432.CCR-15-1294 27037253 10.1158/1078-0432.CCR-15-1294 PMC4877620

[77] Doran SL, Stevanoví S, Adhikary S et al (2019) T-cell receptor gene therapy for human papillomavirus-associated epithelial cancers: a first-in-human, phase I/II study. J Clin Oncol 37:2759–2768. 10.1200/JCO.18 31408414 10.1200/JCO.18.02424 PMC6800280

[78] Nagarsheth NB, Norberg SM, Sinkoe AL et al (2021) TCR-engineered T cells targeting E7 for patients with metastatic HPV-associated epithelial cancers. Nat Med 27:419–425. 10.1038/s41591-020-01225-1 33558725 10.1038/s41591-020-01225-1 PMC9620481

[79] Lahiri A, Maji A, Potdar PD, et al (2023) Lung cancer immunotherapy: progress, pitfalls, and promises. Mol Cancer 22(1):40. 10.1186/s12943-023-01740-y. 36810079 10.1186/s12943-023-01740-y PMC9942077

[80] Park JS, Hur S-Y, Lim MC et al (2021) Efficacy and safety results of GX-188E, a therapeutic DNA vaccine, combined with pembrolizumab administration in patients with HPV 16- and/or 18- positive advanced cervical cancer: phase II interim analysis results (KEYNOTE-567). J Clin Oncol 39:5511–5511. 10.1200/JCO.2021.39.15_suppl.5511

